# Inhibition of ABL1 tyrosine kinase reduces HTLV-1 proviral loads in peripheral blood mononuclear cells from patients with HTLV-1-associated myelopathy/tropical spastic paraparesis

**DOI:** 10.1371/journal.pntd.0008361

**Published:** 2020-07-15

**Authors:** Daisuke Kodama, Masakazu Tanaka, Toshio Matsuzaki, Kimiko Izumo, Nobuaki Nakano, Eiji Matsuura, Mineki Saito, Masahiro Nagai, Masahisa Horiuchi, Atae Utsunomiya, Hiroshi Takashima, Ryuji Kubota, Shuji Izumo

**Affiliations:** 1 Division of Neuroimmunology, Joint Research Center for Human Retrovirus Infection, Kagoshima University, Kagoshima City, Kagoshima, JAPAN; 2 Medical Corporation Sanshukai Ohkatsu Hospital, Kagoshima City, Kagoshima, JAPAN; 3 Department of Hygiene and Health Promotion Medicine, Graduate School of Medical and Dental Sciences, Kagoshima University, Kagoshima City, Kagoshima, JAPAN; 4 Department of Hematology, Imamura General Hospital, Kagoshima City, Kagoshima, JAPAN; 5 Department of Neurology and Geriatrics, Graduate School of Medical and Dental Sciences, Kagoshima University, Kagoshima City, Kagoshima, JAPAN; 6 Department of Microbiology, Kawasaki Medical School, Kurashiki City, Okayama, JAPAN; 7 Department of Neurology and Clinical Pharmacology, Ehime University Graduate School of Medicine, Toon City, Ehime, JAPAN; CSIR-Indian Institute of Chemical Biology, INDIA

## Abstract

Human T-cell leukemia virus type 1 (HTLV-1) causes incurable adult T-cell leukemia and HTLV-1-associated myelopathy/tropical spastic paraparesis (HAM/TSP). Patients with HAM/TSP have increased levels of HTLV-1-infected cells compared with asymptomatic HTLV-1 carriers. However, the roles of cellular genes in HTLV-1-infected CD4+ T cells await discovery. We performed microarray analysis of CD4+ T cells from HAM/TSP patients and found that the *ABL1* is an important gene in HAM/TSP. *ABL1* is a known survival factor for T- and B-lymphocytes and is part of the fused gene (*BCR-ABL*) known to be responsible for chronic myelogenous leukemia (CML). ABL1 tyrosine kinase inhibitors (TKIs), including imatinib, nilotinib, and dasatinib, are used clinically for treating CML. To evaluate whether *ABL1* is indeed important for HAM/TSP, we investigated the effect of TKIs on HTLV-1-infected cells. We developed a propidium monoazide-HTLV-1 viability quantitative PCR assay, which distinguishes DNA from live cells and dead cells. Using this method, we were able to measure the HTLV-1 proviral load (PVL) in live cells alone when peripheral blood mononuclear cells (PBMCs) from HAM/TSP cases were treated with TKIs. Treating the PBMCs with nilotinib or dasatinib induced significant reductions in PVL (21.0% and 17.5%, respectively) in live cells. Furthermore, *ABL1* siRNA transfection reduced cell viability in HTLV-1-infected cell lines, but not in uninfected cell lines. A retrospective survey based on our clinical records found a rare case of HAM/TSP who also suffered from CML. The patient showed an 84.2% PVL reduction after CML treatment with imatinib. We conclude that inhibiting the ABL1 tyrosine kinase specifically reduced the PVL in PBMCs from patients with HAM/TSP, suggesting that *ABL1* is an important gene for the survival of HTLV-1-infected cells and that TKIs may be potential therapeutic agents for HAM/TSP.

## Author summary

Human T-cell leukemia virus type 1 (HTLV-1) is integrated as a provirus in the genomic DNA mainly of CD4+ T cell population in the infected people. HTLV-1-infected CD4+ T cells are transmitted via breast milk, semen, and blood transfusions. HTLV-1 is endemic in Japan, the Middle East, Africa, Caribbean islands, and Central and South America. A small proportion of infected people develop adult T-cell leukemia, HTLV-1-associated myelopathy/tropical spastic paraparesis (HAM/TSP), and other diseases. HAM/TSP, a chronic neuroinflammatory disorder, is characterized by spastic paraparesis and urinary disturbance. HTLV-1-infected CD4+ T cells infiltrate the spinal cord and cause inflammation, which results in such neurological symptoms. We have identified the tyrosine kinase gene *ABL1* as a gene frequently found in the signal transduction pathways in HTLV-1-infected CD4+ T cells. Therefore, *ABL1* appears to be important in the pathogenesis of HAM/TSP. Inhibiting ABL1 with tyrosine kinase inhibitors (TKIs), which is used for chronic myelogenous leukemia (CML), reduced the proviral load (PVL) *in vitro*. We found a rare case of a patient with HAM/TSP and CML by our clinical records, who showed a decrease in PVL after TKI treatment for CML. Hence, TKIs are potential therapeutic agents for HAM/TSP.

## Introduction

Human T-cell leukemia virus type 1 (HTLV-1) mainly infects CD4+ T cells in which its virus genome is integrated into the genomic DNA of the cells. There are 1.08 million infected individuals in Japan, and 5–10 million worldwide. Most of them are asymptomatic HTLV-1 carriers (ACs), but occasionally, some individuals develop adult T-cell leukemia, HTLV-1-associated myelopathy/tropical spastic paraparesis (HAM/TSP), HTLV-1-associated uveitis, and other related diseases [1–5]. The HTLV-1 genome includes *tax*, a regulatory gene coded in the plus strand, and *hbz*, a transcription factor coded in the minus strand. Both viral genes have been investigated in relation to HTLV-1-related diseases [6,7]. HAM/TSP is a neuroinflammatory disease characterized by spastic paraparesis as well as urinary disturbance caused by the inflammation of the spinal cord induced by infiltration of HTLV-1-infected CD4+ T cells [8–10]. Initial studies of HAM/TSP have revealed no disease-specific sequences of HTLV-1 causative of the disease, but a subtype of HTLV-1 partially increases the risk of this disease [11,12]. Many of the risk factors in terms of susceptible genes and the restricting factors for HAM/TSP were identified by investigations of cellular genes, but the most important risk factor for HAM/TSP is an increased HTLV-1 proviral load (PVL) in the peripheral blood [13–16]. The PVL reflects the number of HTLV-1-infected cells in it. In the spinal cords of patients with HAM/TSP, HTLV-1-infected CD4+ T cells were found [8–10,17]. Therefore, reducing the PVL is an essential part of the treatment for HAM/TSP. A recent clinical trial of an anti-CCR4 antibody against HAM/TSP is in progress [18]. However, several clinical studies, including such antibody therapy, are far from the complete control of HTLV-1-infection in HAM/TSP.

In the present study, we investigated the cellular gene expression profiles using a whole human genome DNA microarray with CD4+ T cells (the major *in vivo* reservoir of HTLV-1), from patients with HAM/TSP, AC, or negative controls (NCs). By combining array data processing to refine the differentially expressed genes (DEGs) and pathway analysis, we searched the significant pathways and genes for HAM/TSP.

Herein, our data suggest that *ABL1* gene may play an important role in HAM/TSP and that inhibition of ABL1 tyrosine kinase with TKIs reduces the PVL. These indicate that TKIs, which are known as agents for CML treatment, are potential therapeutic agents for HAM/TSP.

## Materials and methods

### Subjects

The diagnosis of NCs was made when serum anti-HTLV-1 antibody was negative (less than 16×) by particle agglutination (PA) method [19]. Diagnosis of HAM/TSP was made according to the World Health Organization criteria by neurologists belonging to the Department of Neurology and Geriatrics of Kagoshima University Hospital. Subjects who were positive for anti-HTLV-1 antibody but had no neurological symptoms were defined as ACs. We used cryopreserved peripheral blood mononuclear cell (PBMC) samples for microarray analysis from four patients with HAM/TSP, four ACs, and four NCs, from whom we obtained written informed consent. The Ethics Committee of Kagoshima University Hospital approved this study. The statistics of the subjects are summarized in Table 1. There was no significant difference in age among three groups by ANOVA (*P* = 0.801), and the mean PVL of the HAM/TSP group was significantly higher than that of the AC group by Mann-Whitney U test (*P* = 0.021). We randomly chose samples for the experiment, and there occurred a difference in sex.

**Table 1:** Summary of subjects for microarray analysis.

	NC	AC	HAM/TSP
Number	4	4	4
Sex (male: female)	2: 2	1: 3	2: 2
Age (mean ± SD) (y.o.)	52.0 ± 13.9	52.3 ± 4.9	60.0 ± 7.4
Proviral load (copies/10^4^ PBMC)	N/A*	325.8 ± 155.3	2066.0 ± 403.0**

*N/A: not applicable.

**: Significant difference in comparison between HAM/TSP and AC by Mann-Whitney U test (*P* = 0.021). NC: negative control. AC: asymptomatic HTLV-1 carrier. HAM/TSP: HTLV-1-associated myelopathy/tropical spastic paraparesis. SD: standard deviation. y.o.: years old.

### Microarray experiment

CD4+ T cells were separated from PBMCs using the Human CD4+ T cell Isolation Kit (Miltenyi Biotech KK., Tokyo, JAPAN). Total RNA from these cells was extracted. After quality checking by an Agilent Bioanalyzer (Agilent Technologies, Santa Clara, CA, U.S.A.), 50 μg of total RNA was pre-amplified and labeled with cyanine 3-CTP. Complementary DNA was hybridized onto the G4112F Whole Human Genome cDNA microarray 44K × 4plex ver.1.0 (Agilent Technologies). After hybridization, the raw images were acquired and converted into fluorescence intensity data. The data were imported to GeneSpring GX Software ver. 7.3.1 (Agilent Technologies).

### Array data processing and refinement of the genes

Following the Agilent 1-color manual instructions (Agilent Technologies), the raw signal intensities below 5.0 were set to 5.0. Then all the signal intensities were log2 transformed and normalized. To compare the array data from the three groups (NCs, ACs, and HAM/TSP cases), we performed one-way ANOVA at a significant level of *P* < 0.01. Among the significant genes, we extracted significant DEGs that were up-regulated higher than or down-regulated less than 2-fold change when compared with NCs. There are no clear criteria to decide the threshold for fold change in the analysis of array data. We used 2-fold according to previous studies [20]. Then we analyzed the DEGs by cluster analysis to create heat maps, and those DEGs were assigned to a Venn diagram to identify specific cellular genes for HAM/TSP. The raw data of the microarray experiment was deposited to the Gene Expression Omnibus repository at National Center for Biotechnology Information (GSE132666).

### Pathway analysis

The specific cellular genes for HAM/TSP were mapped to the TRANSPATH database (BIOBASE GmbH, Wolfenbuettel, Germany) by ExPlain software (BIOBASE). Pathway analysis was performed using a 2 × 2 contingency table between HAM/TSP and non-HAM/TSP (AC and NC). We extracted the significant pathways for the specific cellular genes. To obtain genes that more frequently appeared than by chance among the specific pathways, we performed a chi-square test or a Fisher’s exact test using a 2 × 2 contingency table for each case by the numbers of genes included in the specific pathways. Then we performed an upstream analysis (BIOBASE). From the sequences in the promoter windows (-1000 to 100 bases from the transcriptional start sites) of the DEGs with more than 2-fold change (Yes-set) and less than 1.1-fold change (No-set), the weighted consensus sequences were calculated, and a transcriptional factor binding site search was performed using the TRANSFAC transcriptional factor binding sites database (BIOBASE) [21]. The results were extracted by the condition of the frequency ratio of the transcriptional binding sites in the Yes-site/No-site > 1.5, Fisher’s exact test (*P* < 0.05) for the number of transcriptional binding sites in the Yes-set, and the Fisher’s exact test (*P* < 0.05) of the matched promoters and binding sites.

### Quantitative reverse transcriptase PCR for *ABL1* mRNA

To evaluate the expression level of the *ABL1* mRNA in enriched HTLV-1-infected cells, we performed real-time quantitative PCR (qPCR) on *ABL1* mRNA in triplicate using the comparative Ct method [22]. Total RNA was extracted from 5×10^6^ CD4+ T cells from 32 individuals, including NCs (N = 7), ACs (N = 14), and HAM/TSP patients (N = 11), and 1 μl of total RNA was transcribed into cDNA. Primer sequences were as follows: *ABL1vβ* mRNA forward, 5’-GCCTGCCCTGCATTTTATC-3’; and reverse, 5’-ATGCTACTGGCCGCTGAA-3’; *GAPDH* mRNA transcript variant 2 forward, 5’-GACTAACCCTGCGCTCCTG-3’; and reverse, 5’-GCCCAATACGACCAAATCAG-3’. *GAPDH* was used as an internal control. cDNA (100 ng) was amplified with 25 μl of Power SYBR Green Master Mix (Applied Biosystems, Foster City, CA, U.S.A.) and 200 nM each of the *ABL1vβ* mRNA sense and antisense primers. Samples and calibrator (Jurkat cell cDNA 100 ng) were subjected to the PCR on the StepOnePlus Real-time PCR system (Applied Biosystems). The amplification cycles were as follows: 50°C, 2 min; 95°C, 10 min; 40 cycles of 95°C, 15 sec, and 60°C for 1 min. The level of *ABL1* mRNA in the query sample was calculated relative to the calibrator standard by the comparative Ct method [22]. The calibrator contained 100 ng of cDNA of the Jurkat cell line at 99.0% viability. To compare the relative mean expression levels of *ABL1* mRNA, we performed a one-way ANOVA and Student’s t-test (significance level, *P* < 0.05) for both tests.

### Cell viability

C91/PL [23] and MT-2 [24] are HTLV-1-infected human T-cell lines, and Molt-4 [25] and Jurkat [26] are uninfected human T-cell lines. We also prepared PBMCs from a patient with HAM/TSP. Those cells were seeded in triplicate onto 96-well plates at a density of 5×10^3^ cells/well. After 48 h cultivation in RPMI 1640 medium with various concentrations of imatinib, nilotinib, and dasatinib, viable cells were counted using XTT Cell Proliferation Kit II (Roche Diagnostics K.K., Tokyo, Japan). Cell viabilities were analyzed in a spectrophotometer and were estimated using the following formula:

Cellviability(%)=(LivecellnumbertreatedwithTKI)/(LivecellnumbertreatedwithoutTKI)×100

### PMA pretreatment of the PBMCs treated with TKIs

Cryopreserved PBMCs from six patients with HAM/TSP were thawed. The cell concentrations were counted by the trypan blue exclusion method. PBMCs at 95.0% viability or higher were adjusted to 1×10^6^ cells/ml and incubated in 2 ml of RPMI 1640 with or without 500 nM nilotinib or 100 nM dasatinib in a 12-well polystyrene dish for 48 h in a 5% CO_2_ incubator. A control well was prepared for each PBMC sample in the same condition without TKI but with 0.006% dimethyl sulfoxide (DMSO), the concentration of which was the same as that in the TKI-treated wells. Each PBMC from the same patient with HAM/TSP were treated with the control (DMSO), nilotinib, or dasatinib. After 48 h, cells were harvested and divided into two. Each of the half samples was pretreated with 50 μM of propidium monoazide (PMA: 3-Amino-8-azido-5-{3-[diethyl(methyl)ammonio]propyl}-6-Phenylphenanthridinium) (Biotium Inc., Fremont, CA, U.S.A.) for 5 min, then photo-crosslinked by exposure to halogen lamp. Each of the other half samples was not pretreated. After that, genomic DNA from all the samples was extracted. PMA is a membrane-impermeable nucleic acid-intercalating fluorescent dye and forms covalent bond by halogen lamp exposure. In PCR with PMA pretreatment, DNA from dead or dying cells is intercalated and is not amplified. PMA pretreatment was performed to quantify *tax* gene numbers only in the live cells.

### Conventional real-time PCR for quantitation of HTLV-1 PVLs

HTLV-1 carries the *tax* gene in the *pX* region of its genome [27]. Using 20 ng of DNA from samples not treated with PMA, we performed real-time qPCR to calculate the HTLV-1 PVL using TaqMan probes and primers for the *tax* gene and *β-actin* (*ACTB*, internal control) as previously described [16]. Real-time qPCR was performed in triplicate with a 25 μl volume for each measurement, using TaqMan Universal Master Mix II (Thermo Fisher Scientific KK.) and the StepOnePlus Real-time PCR system (Applied Biosystems). The cycle threshold was automatically defined by the thermal cycler. Thermal cycling involved one cycle of 50°C for 2 min and 95°C for 10 min; and 45 cycles of 95°C for 15 sec, and 60°C for 1 min. PVL was given as: PVL = (HTLV-1 *tax* amount)/((*β-actin* amount)/2)×10^4^ (copies/10^4^ cells)

### PMA-HTLV-1 viability qPCR for PVL in live PBMCs (PVL_live_)

A new technique, PMA viability PCR has been used to detect live microorganisms in the food and environmental sciences field [28–30]. The new PCR amplifies target in the DNA from live cells but not that from dead cells in the mixture condition. To evaluate the effects of the TKI drug candidates on the HTLV-1-infected cells, PBMCs from HAM/TSP patients were treated with or without the drugs, PMA pretreatment was done, and DNA was extracted from the cells. The novel qPCR assay was performed in the same way as conventional qPCR with standardization using standard curves for *tax* and *β-actin*, and we have tentatively designated it as ‘PMA-HTLV-1-viability qPCR’. Amplification was performed in triplicate using DNA (20 ng) from the PMA pretreated samples. The cycle threshold was automatically defined by the thermal cycler. The HTLV-1 PVL in live PBMCs (PVL_live_) is given as:

$$PVL_{live}=\;\left(HTLV‐1\;tax\;amount\right)/((\beta ‐actin\;amount)/2)\times 10^{4}\;\left(copies/10^{4}\;live\;cells\right)$$

### ABL1 tyrosine kinase assay

Using ADP-Glo Kinase Assay Kit (Promega, Madison, WI., U.S.A) and ABL1 Kinase Enzyme System (Promega, Madison, WI., U.S.A) including Ablitide, a specific peptide substrate for ABL1 tyrosine kinase, ABL1 tyrosine kinase assay was performed according to the manufacturer’s protocol. For evaluating the effect of TKIs, cell lysate extracted after 48 h of incubation with TKIs (either of imatinib 5 μM, nilotinib 500 nM, or dasatinib 100 nM) was applied for this assay in a 96-well plate.

### *ABL1* knockdown by siRNA

We performed siRNA experiment for *ABL1* mRNA in HTLV-1-infected and uninfected cell lines. ABL1 HSS100045 siRNA (Thermo Fisher Scientific K.K., Tokyo, Japan), mixture of Line 1 (5’-CCAACUACGACAAGUGGGAGAUGGA-3’) and Line 2 (5’-UCCAUCUCCCAUUUGUCGUAGUUGG-3’) target *ABL1* mRNA variant α and β exon 4 sequence, very close region to the kinase domain of ABL1 tyrosine kinase. HTLV-1-infected cell lines (MT-2, HUT102, and C91/PL) and uninfected cell lines (Molt-4 and CEM) were transfected with either of siRNA, RNAi Negative Control (Thermo Fisher Scientific K.K., Tokyo, Japan), or blank control using 1 μl of Lipofectamine RNAiMAX (Thermo Fisher Scientific K.K., Tokyo, Japan) per 600 μl of Opti-MEM I Reduced Serum Medium (Thermo Fisher Scientific K.K., Tokyo, Japan). Cells treated with 50 pmol/ml of siRNA were plated at a concentration of 1.67×10^6^ cells/ml. After incubation for 48 h at 37°C in a CO_2_ incubator, cells were harvested, and cell viabilities were evaluated by the trypan blue exclusion method. RNA and cell lysate were also prepared from the cells. Transfection efficiency was assayed by RT-PCR. Ten nanograms of cDNA was applied for RT-PCR using primers for *ABL1* mRNA variant *β* (forward: 5’-AAATGGACTGCACCCGAGAG-3’ and reverse: 5’-CTCATGGTCCAGAGGATCGC-3’) and *ACTB* mRNA (forward: 5’-ACACTGTGCCCATCTACGAC-3’ and reverse: 5’-TGGCCATCTCTTGCTCGAA-3’). The gel image was analyzed by Image J software.

### Statistics

All statistical analyses were performed using Statcel ver.4.0 (OMS Publishing, Tokorozawa, Japan) and GraphPad Prism 6J (GraphPad Software, San Diego, CA, U.S.A.).

## Results

### Specific cellular genes for HAM/TSP extracted by microarray analysis

**Fig 1:**
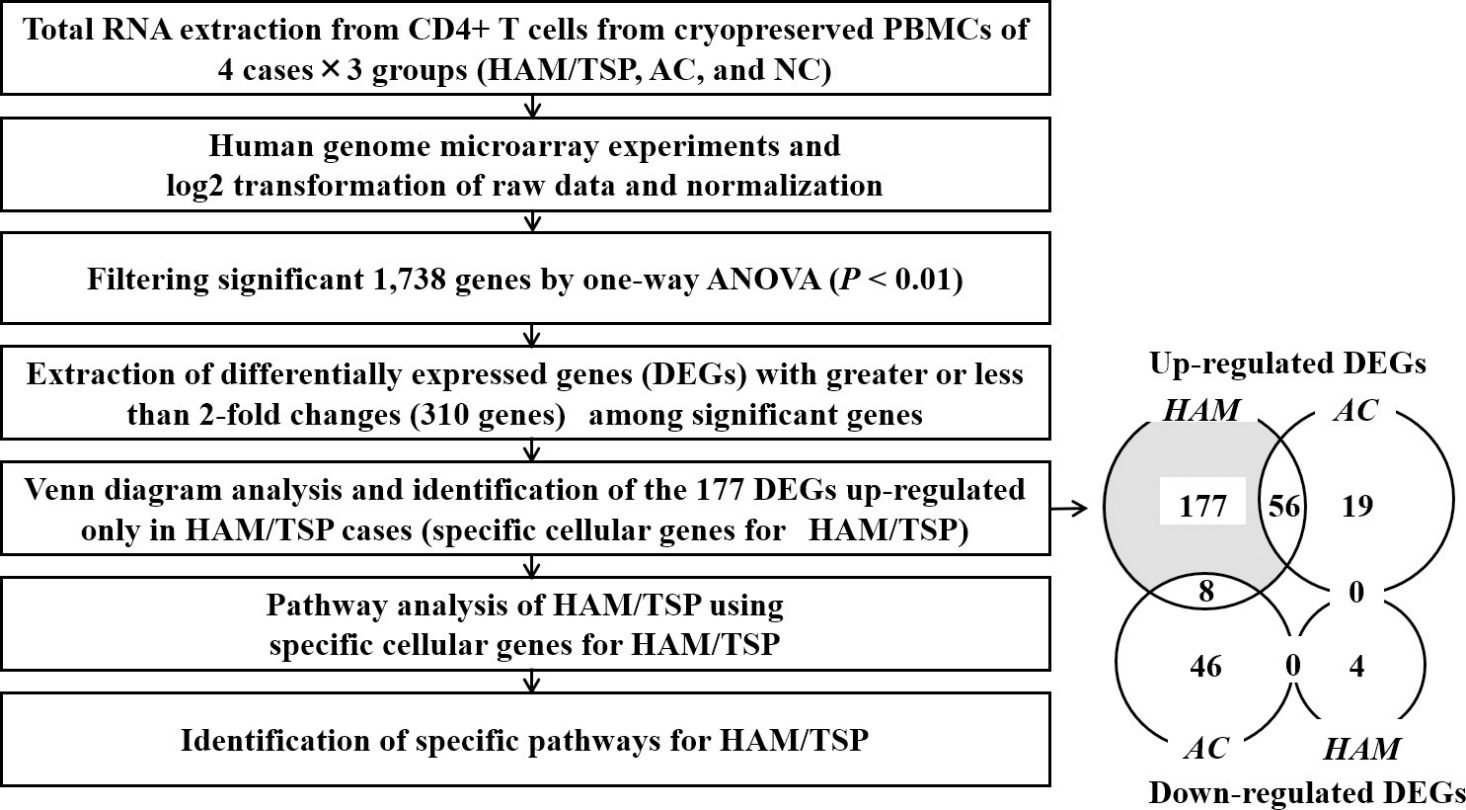
Refinement of specific cellular genes for HAM/TSP. To identify the specific cellular genes and pathways involved in the pathogenesis of HAM/TSP, we performed human genome microarray experiment and data processing according to the presented workflow. Each number in Venn’s diagram represents the number of genes that belong to each area. HAM/TSP: HTLV-1-associated myelopathy, AC: asymptomatic HTLV-1 carrier, NC: negative control.

Through filtering and setting a fold change threshold of DEGs, we extracted 310 genes. By mapping to a Venn diagram, the DEGs were refined to the genes assigned to the area in the diagram where the up-regulated genes and the down-regulated genes belonging to only HAM/TSP. The refined genes were thought to be most important for HAM/TSP. We finally extracted significant 177 DEGs that were up-regulated only in HAM/TSP (Fig 1) and mapped to the shaded area and listed by their fold-change order in Table 2. Pseudogenes, discontinued or replaced genes, and unannotated genes were excluded from the list, resulting in information being available for 109 of the 177 DEGs. As shown in the down-regulated genes list of Table 2, among 4 down-regulated DEGs, 2 of them were not defined, and the remaining 2 were genes whose information was very limited. Therefore, we did not perform a subsequent investigation on these down-regulated genes.

**Table 2:** List of specific genes for HAM/TSP. DEGs up-regulated only in HAM/TSP.

**Gene Symbol**	**Product**	**Accession number**	**Gene ID**	**Relative expression** **(fold change)**	**Function and localization**
*MUC3A*	mucin 3A, cell-surface associated	NM_005960	4584	40.6	Unknown; O-glycosylation in S/T/P repeat
*NAF1*	nuclear assembly factor 1 homolog (S. cerevisiae)	NM_138386	92345	13.7	RNA binding, protein binding
*ZC3H12D*	Zinc finger CCCH-type containing 12D	NM_207360	340152	11.34	Endonuclease activity; regulates macrophage
*POFUT2*	Protein O-fucosyltransferase 2 isoform A	NM_015227	23275	10.71	O-glycosylation in S/T residues in EGF or thrombospondin motifs
*C14orf72*	Chromosome 14 open reading frame	XM_096733	145200	9.73	hypothetical
*CKS2*	CDC28 protein kinase regulatory subunit 2	NM_001827	1164	9.13	Cell cycle, cell proliferation, spindle organization
*SMC4*	Structural maintenance of chromosome 4	NM_005496	10051	7.38	ATP, nucleotide, protein binding, cell cycle, division
*SOX21*	SRY (sex determining region Y)-box 21	NM_007084	11166	6.83	transcription factor activity
*KLHL21*	kelch-like protein 21 (Drosophila)	NM_014851	9903	6.81	Protein binding
*RABGAP1L*	RAB GTPase activating protein 1-like isoform A	NM_014857	9910	6.71	GTPase activator activity; intracellular
*ATP1B1*	Na+/K+ -ATPase beta 1 subunit isoform a	NM_001677	481	6.68	K ion binding; plasma membrane protein
*WDR41*	WD repeat domain 41	NM_018268	55255	6.49	unknown
*TDP1*	tyrosyl-DNA phosphodiesterase 1	NM_018319	55775	6.39	DNA repair, binding
*BCL2L13*	BCL2-like protein 13 (apoptosis facilitator)	NM_015367	23786	6.30	Caspase activator activity
*RPS6KA5*	ribosomal protein S6 kinase, 90kDa, polypeptide 5	NM_004755	9252	6.27	ATP binding; cytoplasm, nucleus
*MAML1*	Mastermind-like [domain containing] 1	NM_005491	9794	5.95	Regulation of transcription; nucleus
*RABEP1*	rabaptin, RAB GTPase binding effector protein 1 isoform 1	NM_004703	9135	5.54	membrane fusion, GTPase activator activity; cytoplasm, endosome
*IL-6ST*	IL-6 signal transducer isoform 1 precursor (gp130, oncostatin M receptor)	NM_002184	3572	5.32	IL-6, -11, -27 receptor activity; positive regulation of T cell proliferation; plasma membrane
*TMEM39A*	Transmembrane protein 9A	NM_018266	55254	5.2	plasma membrane; transmembrane protein
*S100P*	S100 calcium binding protein P	NM_005980	6286	5.06	Calcium and magnesium ion binding; cytoplasm, nucleus
*AFF4*	AFF/FMR2 family, member 4	NM_014423	27125	4.86	Regulation of transcription; nucleus
*SLFN5*	schlafen family, member 5	NM_144975	162394	4.83	ATP, nucleotide binding; cell differentiation; nucleus
*SOD2*	Superoxide dismutase 2, mitochondrial	NM_000636	6648	4.83	Magnesium ion binding; nucleus
*RILPL2*	Rab interacting lysosomal protein-like 2	NM_145058	196383	4.76	Identical protein binding; cytoplasm, cytosol
*PMFBP1*	Polyamine modulated factor 1 (PMF-1) binding protein 1	NM_031293	83449	4.63	Unknown; cytoplasm
*RAN*	Ras related nuclear protein	NM_006325	5901	4.50	GTP binding, GTPase activity, mitotic spindle organization; cytosol, nucleus
*PWP1*	PWP1 homolog (S. cerevisiae), endonuclein	NM_007062	11137	4.32	Transcription; nucleus
*MED28*	Mediator complex subunit 28	NM_025205	80306	4.29	actin binding, protein binding, regulation of transcription; cytoplasm, membrane, nucleus
*HES2*	HES2 (hairy and enhancer of split 2 (Drosophila)) protein, hes family bHLH transcription factor 2	NM_019089	54626	4.17	DNA binding, transcription regulator activity; nucleus
*FAM133B*	family with sequence similarity 133, member B(hypothetical protein LOC257415)	NM_152789	257415	4.14	Unknown
*RRN3*	RNA polymerase I-specific transcription initiation factor RRN3 (TIF-IA)	NM_018427	54700	4.06	Regulation of transcription; nucleoplasm, nucleus
*HERPUD1*	homocysteine-inducible, endoplasmic reticulum stress-inducible, ubiquitin-like domain member 1 protein	NM_014685	9709	4.04	protein binding, response to unfolded protein; ER
*PRPS1L1*	phosphoribosyl pyrophosphate synthetase 1-like 1	NM_175886	221823	3.86	ATP, magnesium ion binding, kinase activity; cellular component
*FBXO3*	F-box protein 3	NM_012175	26273	3.81	ubiquitin-protein ligase activity, proteolysis
*PDCD2*	programmed cell death 2 isoform 1	NM_002598	5134	3.75	DNA, protein, zinc ion binding, apoptosis; cytoplasm, nucleus
*SNAPC5*	small nuclear RNA activating complex, polypeptide 5, 19kDa subunit	NM_006049	10302	3.75	Regulation of transcription; nucleus
*BIRC3*	baculoviral IAP repeat-containing 3 (cIAP-2)	NM_001165	330	3.74	ubiquitin-protein ligase activity, zinc ion binding, anti-apoptosis, signal transduction; cytoplasm, nucleus
*ELN*	elastin	NM_000501	2006	3.71	extracellular matrix constituent; extracellular matrix
*KRT1*	keratin 1	NM_006121	3848	3.7	Protein, sugar binding, receptor activity, cytoskeleton; plasma membrane
*GALNT11*	UDP-N-acetyl-α-D-galactosamine:polypeptide N-acetylgalactosaminyltransferase 11 (GalNAc-T11)	NM_022087	63917	3.69	polypeptide N-acetylgalactosaminyltransferase activity; Golgi
*IKZF5*	IKAROS family zinc finger protein 5	NM_022466	64376	3.65	DNA, zinc ion binding; intracellular, nucleus or NOT nucleus
*TBC1D23*	TBC1 domain family, member 23	NM_018309	55773	3.57	Rab GTPase activator activity; intracellular
*MRPL41*	mitochondrial ribosomal protein L41	NM_032477	64975	3.52	structural constituent of ribosome, apoptosis, cell cycle, translation; mitochondria, ribosome
*NEUROG 3*	neurogenin 3 (class A basic helix-loop-helix protein 7)	NM_020999	50674	3.49	Nervous system development, positive regulation of
*CIRH1A*	cirrhosis, autosomal recessive 1A (cirhin)	NM_032830	84916	3.49	Molecular function; nucleolar, nucleus
*NDUFB3*	NADH dehydrogenase (ubiquinone) 1 beta	NM_002491	4709	3.39	NADH dehydrogenase (ubiquinone) activity, mitochondrial electron transport; mitochondria
*PLA2G4D*	phospholipase A2, group IVD (cytosolic)	NM_178034	283748	3.37	calcium ion binding, hydrolase activity, phospholipase A2 activity; Cytosol, cytoplasm
*PTBP1*	polypyrimidine tract binding protein 1	NM_002819	5725	3.36	RNA splicing; nucleolous, nucleoplasm, nucleus
*HAND1*	heart and neural crest derivatives expressed 1, bHLHa27	NM_004821	9421	3.34	protein heterodimerization activity, transcription; nucleus
*GMCL1*	germ cell-less homolog 1 (Drosophila)	NM_178439	64395	3.34	protein binding, cell differentiation; nucleus
*FAT2*	FAT tumor suppressor homolog 2 (Drosophila)	NM_001447	2196	3.29	calcium ion, protein binding, cell adhesion; plasma membrane
*WARS2*	tryptophanyl tRNA synthetase 2, mitochondrial	NM_201263	10352	3.29	ATP, nucleotide binding, tryptophan-tRNA ligase activity; cytoplasm, mitochondria
*CDX4*	caudal type homeobox transcription factor 4	NM_005193	1046	3.29	Sequence-specific DNA binding, transcription factor; nucleus
*TDG*	thymine-DNA glycosylase	NM_003211	6996	3.27	DNA N-glycosylase activity, damaged DNA, mismatched DNA binding; nucleus
*CCDC85C*	coiled-coil domain containing [protein] 85C	NM_001144995	317762	3.27	Unknown
*ABL1*	c-abl oncogene 1, receptor tyrosine kinase	NM_007313	25	3.26	Apoptosis, cell cycle, cell adhesion, actin cytoskeleton organization, peptidyl-tyrosine phosphorylation, transcription, signal transduction; intracellular
*PRLH*	Prolactin releasing hormone	NM_015893	51052	3.25	Hormone activity; cytoplasm, extracellular
*OASL*	2'-5'-oligoadenylate synthetase-like isoform a	NM_003733	8638	3.24	ATP, DNA, double-stranded RNA binding, thyroid hormone receptor binding, transferase activity; cytoplasm, nucleus
*HCP5*	HLA complex P5	NM_006674	10866	3.23	Defense response; plasma membrane
*CCT6A*	chaperonin containing TCP1, subunit 6A isoform a	NM_001762	908	3.22	ATP, nucleotide, unfolded protein binding; cytoplasm
*SMAD4*	SMAD family member 4	NM_005359	4089	3.16	R-SMAD, collagen binding, BMP signaling; cytosol, cytoplasm, nucleoplasm, nucleus
*SMN2*	survival of motor neuron 2, centromeric	NM_022877	6607	3.15	RNA, protein binding, RNA, mRNA splicing, cell death; cytosol, nucleus
*CCL13*	chemokine (C-C motif) ligand 13	NM_005408	6357	3.14	chemokine activity, signal transducer activity, cell-cell signaling, inflammatory response; plasma membrane
*TRIM23*	tripartite motif-containing 23 (ADP-ribosylation factor domain protein 1 isoform alpha)	NM_001656	373	3.14	GDP, GTP, nucleotide binding; Golgi, cytoplasm
*PFKFB2*	6-phosphofructo-2-kinase/fructose-2, 6-biphosphatase 2 isoform a	NM_006212	5208	3.13	ATP, nucleotide binding, 6-phophofrukto-2-kinase hydrolase, kinase, transferase activity; cytosol
*ERN1*	ER to nucleus signaling 1 isoform 2	NM_152461	2081	3.08	Endonuclease activity, cell cycle arrest, mRNA processing, regulation of transcription; ER, nuclear inner membrane
*GABPB2*	GA binding protein transcription factor, beta subunit 2 isoform beta 1	NM_005254	126626	3.07	protein binding, transcription factor activity; nucleus
*RAPGEF1*	Rap guanine nucleotide exchange factor (GEF) 1 (guanine nucleotide-releasing factor 2 isoform b	NM_198679	2889	3.06	SH3 domain binding, guanyl-nucleotide exchange factor activity, protein binding; cytosol, endosome, intracellular
*YTHDC1*	YTH domain containing protein 1	NM_133370	91746	3.04	RNA splicing, mRNA processing; nucleus
*MORF4L1*	mortality factor 4 like 1	NM_206839	10933	3.03	DNA repair, chromatin modification, histone H2A acetylation, histone H4 acetylation, histone deacetylation; nucleus
*SOX3*	SRY (sex determining region Y)-box 3	NM_005634	6658	3.02	DNA binding; nucleus
*SNRPG*	small nuclear ribonucleoprotein polypeptide G	NM_003096	6637	2.99	RNA binding, RNA splicing, protein binding; cytosol, nucleus
*POLR2K*	DNA directed RNA polymerase II polypeptide	NM_005034	5440	2.98	DNA binding, DNA-directed RNA polymerase activity, zinc ion binding; nucleoplasm, nucleus
*ZMAT2*	zinc finger, matrin type 2	NM_144723	153527	2.97	DNA binding, zinc ion binding; intracellular, nucleus
*AATK*	apoptosis-associated tyrosine kinase	AK131529	9625	2.95	ATP, nucleotide binding, transferase; cytoplasm, mitochondria
*TSPAN13*	Tetraspanin 13 (TM4SF13, NET-6)	NM_014399	27075	2.90	Plasma membrane
*TAAR5*	trace amine associated receptor 5 (putative neurotransmitter receptor)	NM_003967	9038	2.88	G-protein coupled receptor activity; plasma membrane
*TMEM126A*	transmembrane protein 126A	NM_032273	84233	2.87	Molecular function; plasma membrane, mitochondria
*COMP*	cartilage oligomeric matrix protein precursor	NM_000095	1311	2.85	calcium ion binding, ECM structure, cell adhesion; extracellular
*ULBP2*	UL16 binding protein 2	NM_025217	80328	2.80	MHC class I receptor, NK cell activation; plasma membrane
*ADAM7*	a disintegrin and metalloproteinase domain	NM_003817	8756	2.76	metalloendopeptidase activity, zinc ion binding, proteolysis; plasma membrane
*HUS1*	HUS1 checkpoint protein	NM_004507	3364	2.75	protein binding, protein serine/threonine kinase activity, DNA repair, cell cycle; cytoplasm, nucleus
*SH3KBP1*	SH3-domain kinase binding protein 1 (Homo sapiens migration-inducing gene 18 protein)	NM_031892	30011	2.74	SH3 domain binding, protein binding, apoptosis, cell-cell signaling, endocytosis, focal adhesion; plasma membrane
*PPM1B*	protein phosphatase, Mg2+/Mn2+ dependent, 1B	NM_002706	5495	2.7	hydrolase activity, magnesium ion binding, protein binding, protein serine/threonine phosphatase activity
*SMURF1*	SMAD specific E3 ubiquitin protein ligase 1	NM_020429	57154	2.68	ubiquitin-protein ligase activity; cytoplasm, plasma membrane
*SMPX*	small muscle protein, X-linked	NM_014332	23676	2.63	striated muscle contraction; cytoplasm, nucleus
*ADAM21*	ADAM metallopeptidase domain 21 preproprotein	NM_003813	8747	2.63	zinc ion binding, metalloendopeptidase activity, proteolysis, single fertilization; membrane
*IGFALS*	insulin-like growth factor binding protein, acid labile subunit	NM_004970	3483	2.62	IGF binding, protein C-terminus binding, cell adhesion; extracellular region, microsome, soluble fraction
*NPM1*	nucleophosmin (nucleolar phosphoprotein B23, numatrin) 1 isoform 2	NM_199185	4869	2.62	NF-kappaB, Tat protein, RNA, histone binding, protein hetero- or homodimerization, anti-apoptosis, cell-aging
*ZNF407*	zinc finger protein 407 (KIAA1703 protein)	NM_017757	55628	2.61	DNA, zinc ion binding, transcription; intracellular, nucleus
*HLA-E*	MHC, class I, E precursor	NM_005516	3133	2.61	MHC class I receptor activity, antigen processing and presentation; plasma membrane
*MOBKL3*	Mps One Binder kinase activator-like 3 (yeast) (preimplantation protein 3 isoform 1)	NM_015387	25843	2.59	zinc ion, protein binding; Golgi, cytoplasma, membrane
B9D2	B9 protein domain 2 (hypothetical LOC80776)	NM_030578	80776	2.57	cell projection organization; cilium, microtubule basal body, nucleus
*PEX6*	peroxisomal biogenesis factor 6	NM_000287	5190	2.54	ATPase activity, ATP, nucleotide, protein C-terminus binding; cytoplasm, cytosol, peroxisome
*YWHAZ*	tyrosine 3-monooxygenase/tryptophan 5-monooxygenase activation protein, zeta polypeptide	NM_145690	7534	2.52	protein domain specific binding, transcription factor binding, anti- apoptosis; cytoplasm, mitochondria, nucleus
*SUPV3L1*	suppressor of var1, 3-like 1 (S. cerevisiae)	NM_003171	6832	2.49	ATP, DNA, RNA, nucleotide binding, DNA helicase activity, hydrolase activity, DNA duplex unwinding; nucleus, mitochondria
*EIF2S1*	eukaryotic translation initiation factor 2, subunit 1 alpha, 35kDa	NM_004094	1965	2.48	RNA, protein binding, translation initiation factor activity; cytoplasm, cytosol, nucleus
*TUBA1B*	tubulin, alpha 1b	NM_006082	10376	2.48	GTP, protein binding, GTPase activity, microtubule cytoskeleton organization; microtubule
*HNRPCL1*	heterogeneous nuclear ribonucleoprotein C-like 1	NM_0010136	343069	2.48	RNA binding, nucleotide binding; nucleus
*POLB*	polymerase (DNA directed), beta	NM_002690	5423	2.45	DNA-directed DNA polymerase activity, damaged DNA binding, enzyme binding, lyase activity; cytoplasm, nucleoplasm, nucleus, spindle microtubule
*SRPK3*	SFRS protein kinase 3	NM_014370	26576	2.45	ATP, nucleotide, protein binding, protein serine/threonine kinase activity, transferase activity; cellular component
*TSPYL1*	TSPY-like 1	NM_00309	7259	2.44	Molecular function; intracellular, nucleolus, nucleus
*RAI2*	retinoic acid induced 2	NM_021785	10742	2.44	Molecular function, embryonic development; cellular component
*UQCR11*	ubiquinol-cytochrome c reductase, complex III subunit XI	NM_006830	10975	2.44	electron carrier activity, ubiquinol-cytochrome-c reduction activity; mitochondria
*POLDIP3*	polymerase (DNA-directed), delta interacting protein 3	NM_032311	84271	2.41	RNA binding, nucleotide binding, protein binding; nucleus
*RPS14*	ribosomal protein S14	NM_005617	6208	2.38	RNA, mRNA 5'-UTR, protein binding, constituent of ribosome, translation regulator activity; cytosol, nucleolus, ribosome
*SMCHD1*	structural maintenance of chromosomes flexible hinge domain containing 1	NM_015295	23347	2.36	ATP, protein binding; chromosome
*HBS1L*	HBS1(S. cerevisiae) -like translational GTPase	NM_006620	10767	2.32	GTP, nucleotide binding, GTPase activity, translation elongation factor activity
*SLC4A3*	solute carrier family 4, anion exchanger, member 3	NM_001326559	6508	2.22	anion transmembrane transporter activity; inorganic anion exchanger activity; transporter activity; plasma membrane
DEGs down-regulated only in HAM/TSP					
**Gene Symbol**	**Product**	**Accession number**	**Gene ID**	**Relative expression** **(fold change)**	**Function and localization**
N/A	ih08h09.x1 Human insulinoma *Homo sapiens* cDNA 3', mRNA sequence	BM310182.1	N/A	0.30	Unknown. Transcribed locus from EST.
N/A	Homo sapiens cDNA FLJ37957 fis, clone CTONG2009 529	AK095276.1	N/A	0.32	Unknown. hypothetical protein LOC283588.
*MAP7D3*	MAP7 domain containing 3 [*Homo sapiens*]	AL832120	79649	0.36	Member of MAP7 (microtubule-associated protein 7) family. microtubule polymerization and stabilization; cancer growth and metastasis.
*ARFGEF2*	ADP-ribosylation factor guanine nucleotide-exchange factor 2 [*Homo sapiens*]	NM_006420	10564	0.49	Intracellular vesicular trafficking. activation of ARFs by accelerating replacement of bound GDP with GTP and is involved in Golgi transport; guanine-nucleotide exchange activity and also brefeldin A inhibition.

The accession numbers are numbers of the longest variant as representative when there are multiple variants. N/A: not applicable.

### *ABL1* tyrosine kinase gene was suggested as an important gene for HAM/TSP

We extracted 12 significant pathways as specific pathways for HAM/TSP (Table 3). We obtained the frequently-appearing genes in the significant pathways. The *ABL1* tyrosine kinase gene clearly stood out as being commonly included in 11 of the 12 pathways. However, its mRNA expression level was situated in the medium ranking of the up-regulated genes list (Table 2). Upstream analysis of the 177 DEGs did not converge to a few transcription factors (data not shown).

**Table 3:** Specific pathways for HAM/TSP.

Pathway ID of TRANSPATH^a^ database	Pathway name^b^	Group size^c^	Number of hits in group^d^	Number of hits expected^e^	P-value^f^
CH000003804	ABL → TOPBP1	3	2	1	0.00021
CH000000870	ABL → Rad52	4	2	1	0.00043
CH000000972	ABL—/ Bcl-xL	4	2	1	0.00043
CH000003546	ABL → Caspase-9	4	2	1	0.00043
CH000000867	ABL → p73α	5	2	1	0.00071
CH000000908	Caspase-8 → ABL	6	2	1	0.0011
CH000000997	Ubc9 —/ p73α	7	2	1	0.0015
CH000000895	Fas → ABL	10	2	1	0.0031
CH000000977	ABL → p53	11	2	1	0.0038
CH000000869	p73 pathway	24	2	1	0.018
CH000000711	TGFβ pathway	76	3	1	0.026
CH000000879	Caspase network	93	3	1	0.044

a: TRANSPATH is a manually curated database of signal transduction pathways.

b: Pathway name and genes are described as in TRANSPATH. ABL: c-abl oncogene 1, non-receptor tyrosine kinase; TOPBP1: DNA topoisomerase II binding protein 1; RAD52: RAD52 (S. cerevisiae) homolog, DNA repair protein; TP73: tumor protein p73 isoform ɑ; Ubc9: UBE21 (ubiquitin-conjugating enzyme E2I). Symbols in pathway name represent type of reaction (e.g. →: facilitation; —/: inhibition).

c: Group size: the total number of genes, proteins, and metabolites which exist within the pathway of interest.

d: Number of hits in group: number of the cellular genes of interest (e.g. up-regulated DEGs only in HAM/TSP), which exist within the pathway chosen by a statistical test.

e: Number of hits expected: number of the cellular genes of interest that are included in the pathway chosen by statistical test by chance.

f. *P*-value of two-tailed Fisher’s exact test.

### Verification of up-regulation of *ABL1* mRNA by quantitative RT-PCR

**Fig 2:**
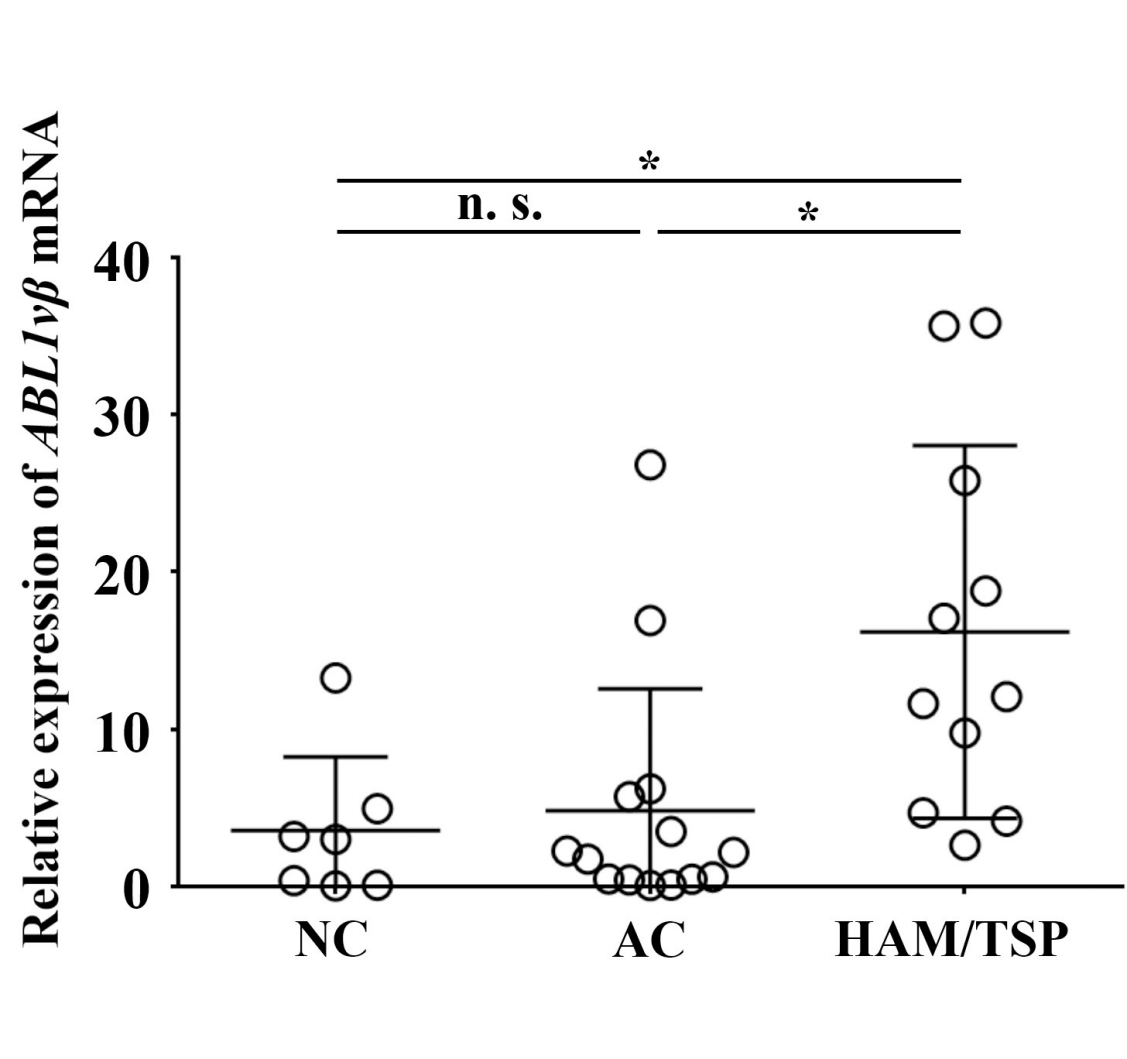
*ABL1* gene expression in CD4+ T cells by quantitative RT-PCR. The total RNA extracted from CD4+ T cells from the subjects including NC (N = 7), AC (N = 14), and HAM/TSP (N = 11) was reverse-transcribed. The relative expression levels of *ABL1vβ* mRNA were calculated by the comparative Ct method, and 100 ng cDNA of Jurkat cell line was used for the calibrator gene. *GAPDH* mRNA was used as an internal control. The relative mean expression level of *ABL1* mRNA was 3.56 for NC, 4.83 for AC, and 16.19 for HAM/TSP group. Student’s t-test was used. Bars represent mean ± standard deviation (SD). *: significant at *P <* 0.05. n.s.: not significant.

To verify the microarray results where the *ABL1* mRNA expression level in CD4+ T cells in the HAM/TSP cases was up-regulated in comparison with ACs or NCs, we performed qRT-PCR. The relative expression level of *ABL1* mRNA was significantly higher in HAM/TSP patients than in NCs (*P* = 0.013) and ACs (*P* = 0.029) by Student’s t-test (Fig 2).

### The effect of TKI treatment on cell viability of HTLV-1-infected cell lines and PBMCs from HAM/TSP patients

**Fig 3:**
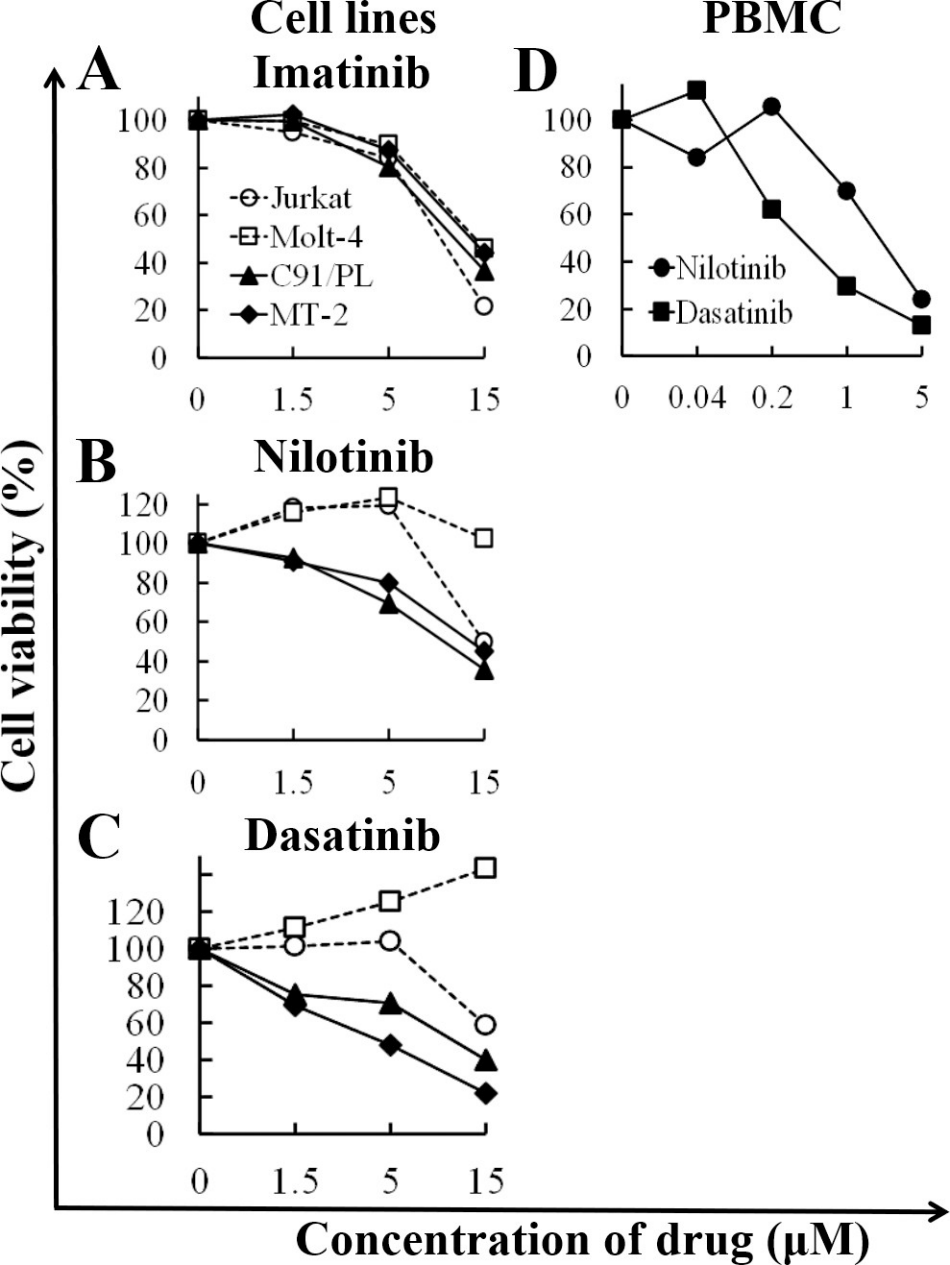
Viability of cell lines and HAM/TSP PBMC treated with ABL1 tyrosine kinase inhibitors (TKIs). HTLV-1-uninfected cell lines (Jurkat and Molt-4), HTLV-1-infected cell lines (C91/PL and MT-2), and PBMC from a HAM/TSP patient were cultured with various concentrations of TKIs for 48 h. Cell viability was measured by an XTT assay in triplicate. A: viability of cell lines treated with imatinib. B: viability of cell lines treated with nilotinib. C: viability of cell lines treated with dasatinib. D: viability of HAM/TSP PBMC treated with TKIs (nilotinib or dasatinib). X- and y-axis show the concentration of TKIs (μM) and cell viability (%), respectively. Cell viability (%) represents the live cell number treated with TKIs relative to that without treatment. Open circle: Jurkat; open square: Molt-4; solid triangle: C91/PL; solid diamond: MT-2; solid circle: nilotinib; and solid square: dasatinib.

To investigate whether inhibition of ABL1 tyrosine kinase has an effect on HTLV-1-infected or uninfected cell lines and PBMCs from HAM/TSP cases, we performed an XTT assay (Fig 3). Both HTLV-1 uninfected cell lines (Jurkat and Molt-4) and HTLV-1-infected cell lines (C91/PL and MT-2) were similarly viable when the cells were treated with various concentrations of imatinib (Fig 3A). However, the HTLV-1-infected cell lines had relatively low viability over a wide range of nilotinib or dasatinib concentrations (Fig 3B and 3C), suggesting that both inhibitors more selectively induced cell death for HTLV-1-infected cells compared with imatinib. Therefore, we chose nilotinib and dasatinib for the next XTT assay to explore the appropriate concentration of each TKI for PBMCs from a patient with HAM/TSP. We performed this assay with concentrations up to 5 μM (Fig 3D), which was the maximum concentration where 100% of the uninfected cell lines survived (Fig 3B and 3C). We estimated 100 nM and 500 nM to be the optimal concentrations at which the PBMCs remained viable at more than 80% for dasatinib and nilotinib, respectively (Fig 3D). Using these data, the IC50 of TKIs for each cell line in the XTT assay was calculated (S1 Table).

### TKIs reduced HTLV-1 PVL in live PBMCs from patients with HAM/TSP

**Fig 4:**
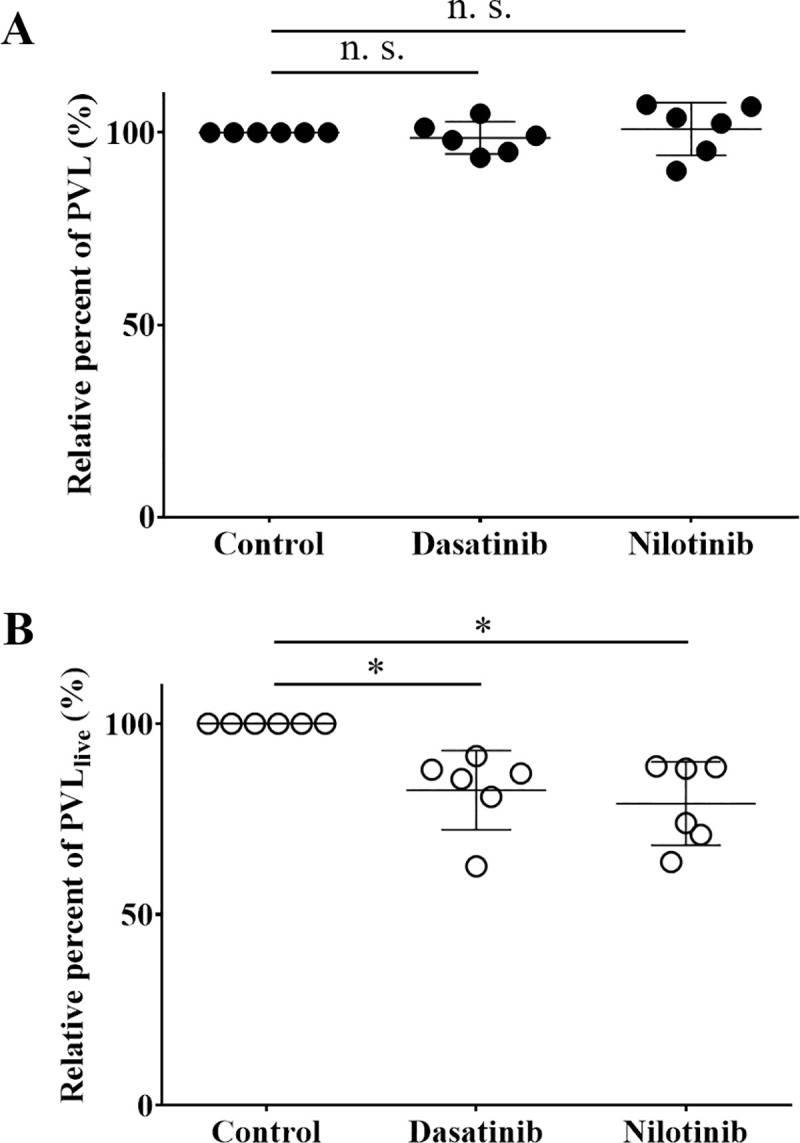
The effect of TKIs to PVL in total PBMC or in live PBMC from HAM /TSP patients. PBMC from patients with HAM/TSP were cultured with 0.006% dimethyl sulfoxide (control), 100 nM dasatinib, or 500 nM nilotinib for 48 h and were harvested. Genomic DNA was extracted from the cells and then subjected to both conventional qPCR for PVL (A) and PMA-HTLV-1 viability qPCR (B). The former counts PVL in total PBMC (a mixture of live and dead cells), while the latter enables the quantification of the PVL only in live PBMC (PVL_live_). A: conventional qPCR for PVL. Solid circles represent PVL relative to that of the control, and bars represent means and SD. There was no significant difference in the relative PVL in the cells treated with or without TKI. B: PMA-HTLV-1 viability qPCR to quantify PVLs only in live cells (PVL_live_). Open circles represent PVL_live_ relative to that of the control, and bars represent means and SD. The mean PVL_live_ of samples treated with dasatinib or nilotinib were significantly reduced at 17.5% and 21.0% relative to the control, respectively. Student’s t-test was used (N = 6). *: significant at *P <* 0.05; n.s.: not significant.

To test whether *ABL1* is an important gene involved survival of HTLV-1-infected cells, we investigated PVL after the treatment of TKIs. By the conventional qPCR, there was no significant difference in the PVL with or without TKI treatments (Fig 4A). Conventional qPCR has a possibility to amplify DNA from dead cells [31]. Therefore, we developed PMA–HTLV-1 viability qPCR to distinguish live and dead cells and evaluated the effect of drugs on HTLV-1-infected cells. To validate PMA-HTLV-1 viability qPCR, we performed this method and conventional qPCR for PVL using mixtures of HUT102 live and dead cells in various ratios (S1 Fig). The Ct values of the two methods were completely coincident for 100% live cells. However, the difference of the Ct values expanded significantly with the increase of dead cell percentage. The result suggested that PMA-HTLV-1 viability PCR was more sensitive for HTLV-1-infected cell death than conventional qPCR. Using this assay, the relative percentage of PVL_**live**_ (%) showed a significant reduction of 17.5% and 21.0% after incubation with either dasatinib or nilotinib, respectively (Fig 4B).

### TKIs suppressed ABL1 tyrosine kinase activity in both HTLV-1-infected or uninfected cell lines

**Fig 5:**
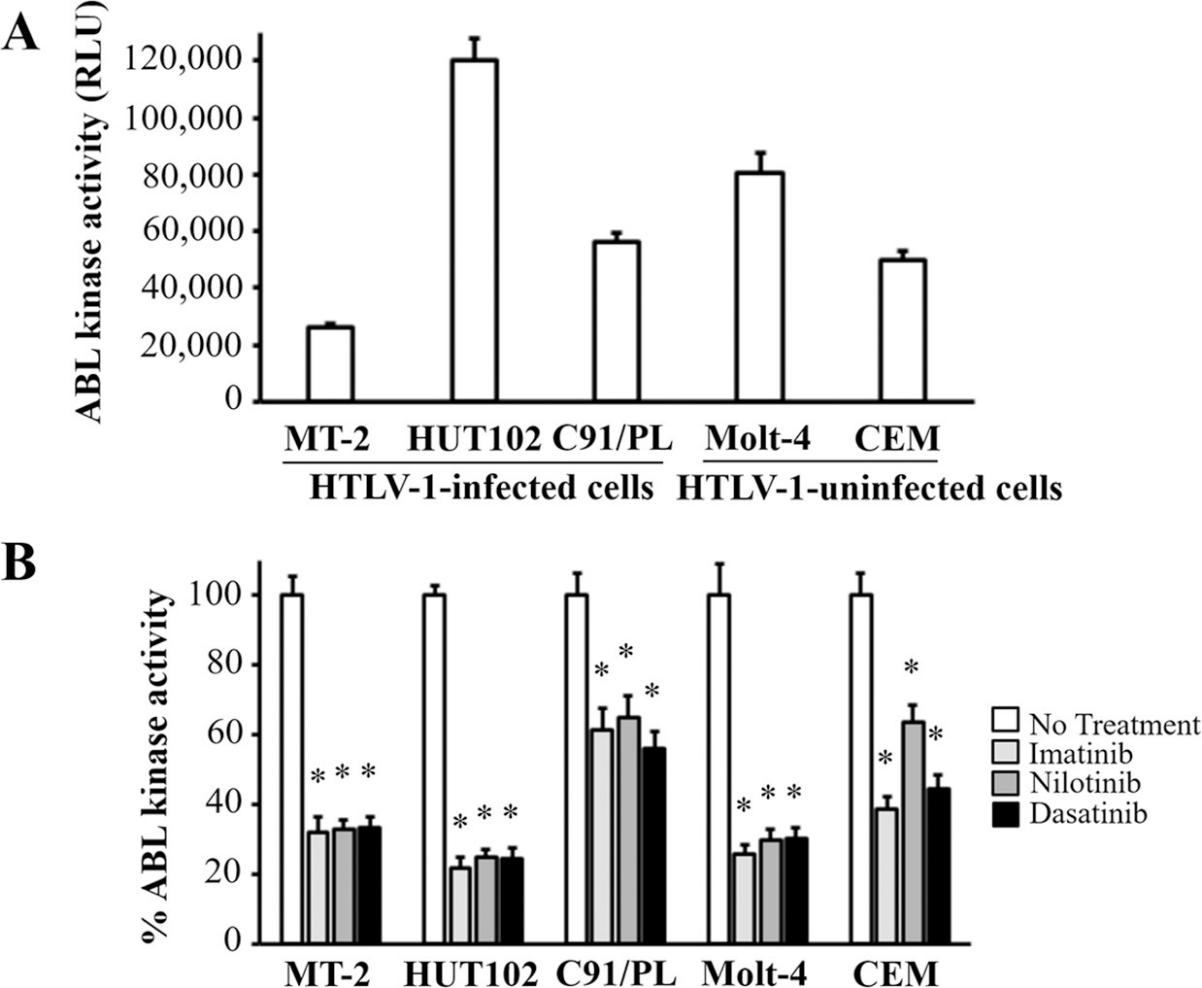
Effect of TKIs on ABL1 tyrosine kinase activity of HTLV-1-infected or uninfected cell lines. A: Basal level of ABL1 tyrosine kinase activity of HTLV-1-infected or uninfected cell lines. B: Relative ABL1 tyrosine kinase activity (%) of cells treated with or without TKIs for 48 h. Error bars represent SD. Asterisks in panel B indicate statistically significant (*P* < 0.05, paired t-test) when compared with that of the cells without treatment (no treatment).

At first, we measured the kinase activities in HTLV-1-infected and uninfected cell lines and found that the kinase activities varied among cell lines (Fig 5A). Next, we investigated the effect of TKIs on kinase activities. We found that ABL1 tyrosine kinase activities in all cell lines treated with TKIs were reduced compared with those of cell lines without treatment (Fig 5B).

### Knockdown of *ABL1* mRNA by siRNA reduced cell viability in HTLV-1-infected cell lines

**Fig 6:**
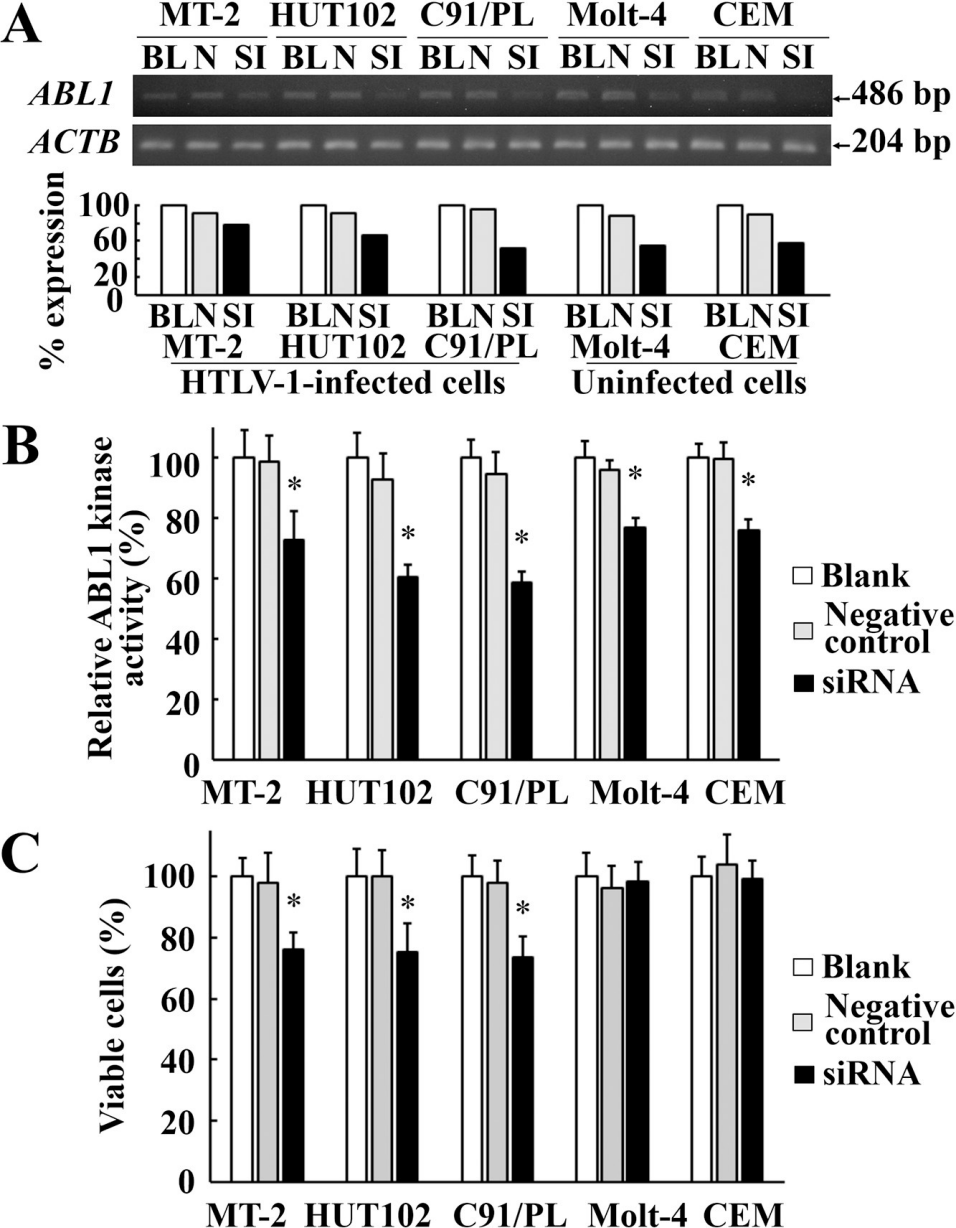
*ABL1* mRNA knockdown by siRNA in HTLV-1-infected or uninfected cell lines. A: RT-PCR of HTLV-1-infected or uninfected cell lines after *ABL1* siRNA transfection. Upper and lower gel images represent amplicon of *ABL1* mRNA and *ACTB* mRNA, respectively. The bar graph under the gel images indicates relative expression of *ABL1* mRNA standardized by *ACTB* mRNA. BL, N, and SI represent blank control, negative control siRNA, and *ABL1* siRNA, respectively. B: Relative ABL1 tyrosine kinase activities of HTLV-1-infected or uninfected cell lines after *ABL1* siRNA transfection. Grey and solid bars indicate the relative ABL1 kinase activities in each cell line treated with negative control siRNA or *ABL1* siRNA to those without siRNA treatment (white bars), respectively. Error bars represent SD. Asterisks indicate statistically significant (*P* < 0.05, paired t-test) when compared with those treated with negative control siRNA (gray bars). C: Cell viabilities of HTLV-1-infected or uninfected cell lines after *ABL1* siRNA transfection. Grey and solid bars represent the relative percent of viable cells in each cell line treated with negative control siRNA or *ABL1* siRNA to those without siRNA treatment (white bars), respectively. Error bars represent SD. Asterisks indicate statistically significant (*P* < 0.05, paired t-test) when compared with those treated with negative control siRNA (gray bars).

To investigate whether TKIs really target ABL1 tyrosine kinase and cause a reduction in HTLV-1 PVL, we performed a siRNA experiment targeting the kinase region of *ABL1*. After *ABL1* siRNA transfection, *ABL1* expression was reduced in HTLV-1-infected cell lines (MT-2, HUT102, and C91/PL) as well as in uninfected cell lines (Molt-4 and CEM) (Fig 6A), and ABL1 tyrosine kinase activities were also reduced in all cell lines (Fig 6B). In a cell viability assay, viable cells were significantly reduced in HTLV-1-infected cell lines, but not in the uninfected cell lines (Fig 6C).

### Presentation of a HAM/TSP case complicated by CML that was treated with imatinib

As a retrospective survey based on our clinical records, we searched for HTLV-1-positive CML cases where the PVL was estimated before and after the treatment of the CML with a TKI. We found only one case who had both CML and HAM/TSP. The patient was a 52-year-old male diagnosed as having HAM/TSP. Osame’s Motor Disability Score (OMDS) was 6/13 for this patient (*i*.*e*., able to walk more than 10 m with a cane and holding on to something) [32]. After prednisolone administration (5 mg/day), the OMDS became 4/13 (*i*.*e*., walking without a cane but a handrail is necessary when climbing steps). Eight years later, he was diagnosed with CML because of leukocytosis, a Philadelphia-positive chromosome, and a low neutrophil alkaline phosphatase score. On admission, the OMDS was 6/13, the PVL was 2,844 copies/10^4^ in PBMCs, and neopterin in the cerebral spinal fluid was 86.2 nmol/ml (the normal value is ≦30). He was administered imatinib (400 mg/day) for CML treatment for the first month, followed by 300 mg/day for 30 months. After imatinib treatment, he showed molecular genetics-based remission of the CML. Additionally, the OMDS improved from 6/13 to 5/13 (*i*.*e*., he was able to walk while holding on to something). Likewise, the PVL decreased from 2,844 to 448 copies/10^4^ PBMCs (an 84.2% reduction). Severe adverse effects by the imatinib treatment were not seen during the whole treatment period.

## Discussion

We identified the ABL1 tyrosine kinase gene as being important in HAM/TSP. The pathway analysis revealed that *ABL1* gene was involved in many of the relevant pathways for HAM/TSP specific genes extracted by the microarray data. The *ABL1* expression level was relatively high in the CD4+ T cells from HAM/TSP patients and ranged in the list for the specific genes for HAM/TSP (Table 2). The finding of the up-regulation of *ABL1* mRNA in HTLV-1-infected cells is supported by a study of other researchers [33]. In addition, we showed that ABL1 tyrosine kinase inhibitors, dasatinib and nilotinib, reduced HTLV-1 PVL in cultured PBMC from the HAM/TSP patients (Fig 4B). In cell viability assay, the TKIs preferentially reduced the viability of HTLV-1-infected cell lines rather than uninfected cell lines (Fig 3A–3C). The addition of TKIs induced the significant reduction in ABL1 kinase activity in both of HTLV-1-infected or uninfected cell lines (Fig 5B). At the same time, siRNA experiment revealed that the knockdown of *ABL1* mRNA induced cell death in HTLV-1-infected cell lines, but not in uninfected cell lines (Fig 6C). These data suggest that ABL1 tyrosine kinase inhibitors reduced PVL and preferentially induced cell death in HTLV-1-infected cells. These data are consistent with the clinical case, who was diagnosed CML in the clinical course of HAM/TSP and showed an 84.2% reduction in PVL after imatinib treatment for CML. These *in vitro* and clinical data suggest that ABL1 tyrosine kinase inhibitors may be therapeutic agents for HAM/TSP.

HAM/TSP patients show higher PVLs compared with ACs [16]. PVL is known to be associated with disease exacerbation [34–36], rapid disease progression, and many aspects of the central nerve system (CNS) inflammation in HAM/TSP [8–10]. These findings strongly indicate that PVL reduction is crucial for improving the neurological dysfunctions in patients with HAM/TSP. For example, treatments with interferon-α or anti-CCR4 antibody are reported to reduce PVLs somewhat [18, 37]. Therefore, another treatment to reduce the PVL, such as TKIs shown in this study, is needed for HAM/TSP.

The three TKIs used in the present study reduced the ABL1 kinase activity in all tested cell lines (Fig 5B). Imatinib reduced cell viability in both HTLV-1-infected (C91/PL and MT-2) and uninfected cell lines (Jurkat and Molt-4), while nilotinib and dasatinib showed a relatively specific reduction of cell viability for HTLV-1-infected cells (Fig 3). These results were similar to previous findings on *in vitro* activity of TKIs, in which nilotinib and dasatinib are more sensitive for ABL1 tyrosine kinase activity compared to imatinib [38]. Nilotinib and dasatinib are blood-brain barrier-permeable, but imatinib is impermeable [39–41]. As a treatment for HAM/TSP, nilotinib and dasatinib may be more appropriate therapeutic agents than imatinib for inducing death of HTLV-1-infected cells infiltrating the spinal cord, which may result in reducing inflammation in the CNS.

TKIs reduced cell viability of HTLV-1infected cell lines (Fig 3) and PVL in PBMC from HAM/TSP patients (Fig 4B). However, it was unclear whether these effects occurred via specific inhibition of ABL1 tyrosine kinase. In our study, the TKIs reduced the ABL1 kinase activities in HTLV-1-infected or uninfected T cell lines (Fig 5B). In addition, in the *ABL1* siRNA experiment targeting ABL1 tyrosine kinase, cell viabilities were significantly reduced in the HTLV-1-infected cell lines, but not in the uninfected cell lines (Fig 6C). Although we cannot completely deny the possibility that death in HTLV-1-infected cells occurred via the off-target effect of the TKIs, these results suggest that TKIs targeted ABL1 tyrosine kinase and induced cell death in HTLV-1-infected cells which showed the increased expression of *ABL1* gene.

QPCR for PVL quantitation is a standard method for estimating HTLV-1 provirus in genomic DNA from PBMC. However, when cells are dead during a short incubation with a drug, it was known that DNA extracted from the dead cells could be amplified by the qPCR [31]. In our experiment, as shown in Fig 4A, conventional PCR did not show any inhibitory effects of TKIs on PVL in cultured PBMC from HAM/TSP patients. Therefore, we developed a novel technique, PMA-HTLV-1 viability qPCR, with PMA pretreatment before amplification. Our preliminary experiment for this method showed apparent differences in Ct values, depending on the ratio of dead cells in samples (**S1 Fig**). When the ratio of dead cells was increased, the Ct value in the case with PMA treatment became high compared to that without PMA treatment, suggesting that the treatment made it possible to evaluate PVL in live cells more sensitive. By applying the method to clinical samples with TKI treatment, we could more accurately evaluate PVL in live cells than the conventional qPCR and found that the TKIs reduced PVL (Fig 4B). Of course, there is a limitation that the PMA-HTLV-1 viability qPCR amplified some of DNA even from 100% dead cells, to a lesser extent compared to the conventional qPCR (**S1 Fig**). Our data suggest that PMA-HTLV-1 viability qPCR can be expanded to an application that quantifies another target in the mixture of live and dead cells.

## Conclusions

We identified the ABL1 tyrosine kinase gene as being important in HAM/TSP by combining microarray and pathway analysis. The addition of the ABL1 tyrosine kinase inhibitors, nilotinib, and dasatinib, into the PBMC culture significantly reduced the PVL in live PBMCs by 21.0% and 17.5%, respectively. The knockdown of *ABL1* mRNA induced cell death in HTLV-1-infected cell lines. We also found a HAM/TSP case who was complicated by CML. He showed an 84.2% reduction in PVL after imatinib treatment. These data suggest that inhibiting ABL1 tyrosine kinase by TKI can reduce HTLV-1 PVL in patients with HAM/TSP.

## Ethics statement

The Ethics Committee of Kagoshima University Hospital, Japan, approved this study. All human subjects were adults except one. We obtained written informed consent from all subjects. We explained to the child about blood sampling and obtained written informed consent from him with his parents. This report does not contain any individual data.

## Supporting information

S1 Table IC50 of TKIs in cell viability assay. (DOCX)

S1 Fig The effect of PMA pretreatment on cycle threshold in evaluating HTLV-1 proviral load. To evaluate whether the mixture of live and dead cells and PMA pretreatment affect HTLV-1 PVL measurement, we induced dead cells by digitonin treatment at 300 ng/ml for 2 h and made five different cell mixture (percent ratios of live /dead cells were 100/0, 75/25, 50/50, 25/75, and 0/100) using HTLV-1-infected cell line HUT102. We divided these cell mixtures into two groups: one was pretreated with PMA (which enters only dead cells and fix the DNA), and the other was not. Genomic DNA was extracted and we performed quantitative PCR targeting HTLV-1 *pX* gene in triplicate and repeated twice. Error bars represent standard error of the mean.
(TIF)
