# Supplementary material for: Inhibition of ABL1 tyrosine kinase reduces HTLV-1 proviral loads in peripheral blood mononuclear cells from patients with HTLV-1-associated myelopathy/tropical spastic paraparesis
Source: PLoS Negl Trop Dis. 2020 Jul 15;14(7):e0008361. doi: 10.1371/journal.pntd.0008361 (PMC7363079; doi:10.1371/journal.pntd.0008361)
Supplement: S1 Table — (DOCX) [file pntd.0008361.s001.docx]

**S1 Table. IC50 of TKIs in cell viability assay.**

| Cell | Imatinib | Nilotinib | Dasatinib |
| --- | --- | --- | --- |
| MT-2 | 12.86 | 12.88 | 10.52 |
| C91/PL | 10.66 | 9.49 | 4.45 |
| Molt-4 | 13.60 | N. A. | N. A. |
| Jurkat | 9.09 | 14.93 | N. A. |
| PBMC | N. E. | 2.00 | 0.36 |

N.A.: not applicable. N. E.: not examined. Numbers represent IC50 (μM).

The table shows IC50 calculated from cell viability shown in Fig.3.
