# Supplementary figures and images for: Inhibition of ABL1 tyrosine kinase reduces HTLV-1 proviral loads in peripheral blood mononuclear cells from patients with HTLV-1-associated myelopathy/tropical spastic paraparesis
Source: PLoS Negl Trop Dis. 2020 Jul 15;14(7):e0008361. doi: 10.1371/journal.pntd.0008361 (PMC7363079; doi:10.1371/journal.pntd.0008361)

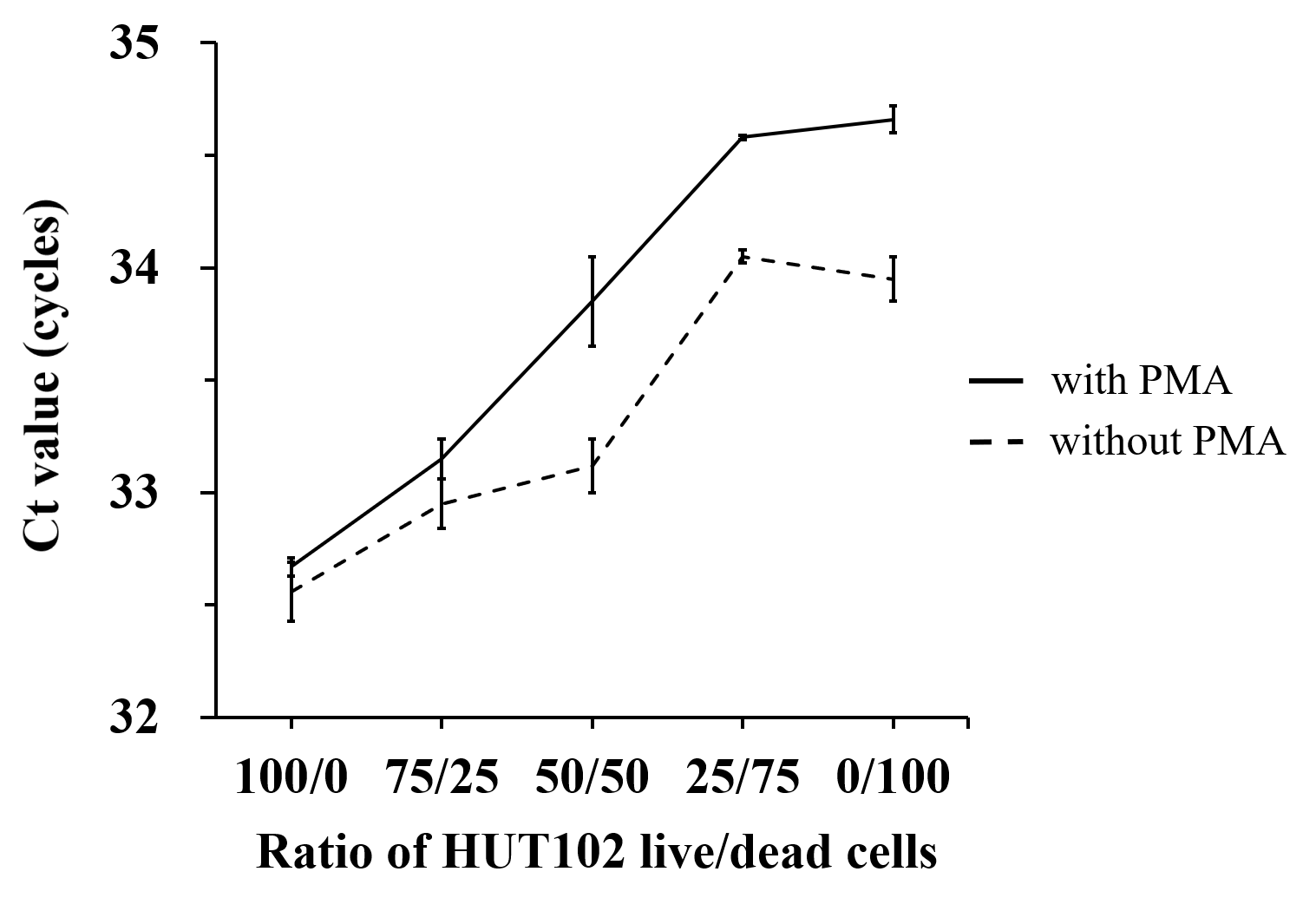

Supplement: S1 Fig — To evaluate whether the mixture of live and dead cells and PMA pretreatment affect HTLV-1 PVL measurement, we induced dead cells by digitonin treatment at 300 ng/ml for 2 h and made five different cell mixture (percent ratios of live /dead cells were 100/0, 75/25, 50/50, 25/75, and 0/100) using HTLV-1-infected cell line HUT102. We divided these cell mixtures into two groups: one was pretreated with PMA (which enters only dead cells and fix the DNA), and the other was not. Genomic DNA was extracted and we performed quantitative PCR targeting HTLV-1 pX gene in triplicate and repeated twice. Error bars represent standard error of the mean. (TIF) [file pntd.0008361.s002.tif]
